# Prevalence and correlates of chronic obstructive pulmonary disease and chronic respiratory symptoms in rural southwestern Uganda: a cross-sectional, population-based study

**DOI:** 10.7189/jogh.09.010434

**Published:** 2019-06

**Authors:** Crystal M North, Bernard Kakuhikire, Dagmar Vořechovská, Simone Hausammann-Kigozi, Amy Q McDonough, Jordan Downey, David C Christiani, Alexander C Tsai, Mark J Siedner

**Affiliations:** 1 Massachusetts General Hospital, Boston, Massachusetts, USA; 2 Harvard Medical School, Boston, Massachusetts, USA; 3 Harvard T.H. Chan School of Public Health, Boston, Massachusetts, USA; 4 Mbarara University of Science and Technology, Mbarara, Uganda; 5 Last Mile Health, Cambridge, Massachusetts, USA

## Abstract

**Background:**

The global burden of chronic obstructive pulmonary disease (COPD) disproportionately affects resource-limited settings such as sub-Saharan Africa (SSA), but population-based prevalence estimates in SSA are rare. We aimed to estimate the population prevalence of COPD and chronic respiratory symptoms in rural southwestern Uganda.

**Methods:**

Adults at least 18 years of age who participated in a population-wide census in rural southwestern Uganda completed respiratory questionnaires and lung function testing with bronchodilator challenge at health screening events in June 2015. We defined COPD as post-bronchodilator forced expiratory volume in one second to forced vital capacity ratio less than the lower limit of normal. We fit multivariable linear and log binomial regression models to estimate correlates of abnormal lung function and respiratory symptoms, respectively. We included inverse probability of sampling weights in models to facilitate population-level estimates.

**Results:**

Forty-six percent of census participants (843/1814) completed respiratory questionnaires and spirometry, of which 565 (67%) met acceptability standards. COPD and respiratory symptom population prevalence were 2% (95% confidence interval (CI)  = 1%-3%) and 30% (95% CI = 25%-36%), respectively. Respiratory symptoms were more prevalent and lung function was lower among women and ever-smokers (*P* < 0.05). HIV serostatus was associated with neither respiratory symptoms nor lung function.

**Conclusions:**

COPD population prevalence was low despite prevalent respiratory symptoms. This work adds to the growing body of literature depicting lower-than-expected COPD prevalence estimates in SSA and raises questions about whether the high respiratory symptom burden in rural southwestern Uganda represents underlying structural lung disease not identified by screening spirometry.

Over one billion people worldwide suffer from chronic respiratory diseases. Chronic obstructive pulmonary disease (COPD) is the third leading cause of death and disability globally [1] and has substantial negative impacts on work performance and economic productivity [2]. According to the Global Burden of Disease report, COPD is estimated to be among the top ten causes of death in low and middle income countries [1], and modeled estimates based on data through 2010 indicate that over 26 million people are living with COPD in sub-Saharan Africa (SSA) alone [3] – nearly as many as are living with HIV [4]. However, these modeled estimates are based on a limited amount of data from few countries and employ a variety of survey methodologies. Thus, uncertainty remains regarding their accuracy.

Population-based estimates of COPD prevalence in SSA are rare. Cohort studies in SSA have yielded prevalence estimates ranging from 4%-25% depending on the age distribution and prevalence of COPD-associated risk factors in the studied population [3,5], and few studies have used spirometry-based diagnostics or population-based sampling techniques. The only spirometry-based COPD prevalence estimates in Uganda report prevalence estimates ranging from 1.7% to 16% [6,7], and there are no prevalence estimates of chronic respiratory symptoms. The prevalence of risk factors for impaired lung function – including air pollution and tobacco exposure, tuberculosis and other lower respiratory tract infections, HIV infection and malnutrition – are different in SSA compared to high income settings, and this differential epidemiology is likely to influence chronic lung disease prevalence [8]. As such, limited data are available to guide regional public health officials in identifying at-risk populations. We conducted spirometry with bronchodilator testing at health screening events in rural southwestern Uganda to estimate the population prevalence of COPD and chronic respiratory symptoms.

## METHODS

This cross-sectional, population-based study was conducted in Nyakabare Parish, a rural community within Mbarara district, southwestern Uganda. Most residents of Mbarara district practice subsistence farming [9] and live in rural settings where both food and water insecurity are common [10,11].

Most eligible adults in Nyakabare Parish (98%, 1814/1851) were enumerated in a population census conducted from 2014 to 2015. The census sought to enroll all adults aged 18 years and older (and emancipated minors aged 16 to 18 years) who reported having stable, primary residence in Nyakabare Parish. There were no other inclusion or exclusion criteria. Adults at least 18 years of age who participated in the population census were invited to attend one of five health screenings in June 2015 (Figure S1 in **Online Supplementary Document**), and those who attended were eligible for study participation. The health screenings were advertised through community meetings, printed advertisements, and radio and church announcements. Free transportation was arranged for community members with poor health or limited mobility.

Trained study staff collected demographic and health information with structured questionnaires (Figure S2 in **Online Supplementary Document**). Study staff included research assistants who administered study questionnaires, phlebotomists who collected blood samples, and medical professionals and students who conducted the medical screenings. Smoking history was collected with a modified WHO STEPS questionnaire [12]. Participants were identified as having a chronic cough and/or chronic dyspnea if they responded “yes” to the following questions: 1) “Do you have a cough on most days?” and/or 2) “Do you have difficulty in breathing on most days?” Participants who reported a cough were asked about sputum production. Body mass index was calculated by dividing weight (in kilograms) by height squared (in centimeters). Confidential HIV testing was offered in accordance with national guidelines [13].

Trained volunteers measured forced expiratory volume in one second (FEV_1_) and forced vital capacity (FVC) with EasyOne® Plus handheld spirometers (nDD Medical Technologies, Andover, USA) in accordance with American Thoracic Society (ATS) standards [14]. Spirometers were factory-calibrated prior to use, and multi-flow calibration was confirmed using a 3-L syringe after the final day of study procedures. All subjects had refrained from smoking for at least one hour prior to testing. Four puffs of albuterol (Ventolin, GlaxoSmithKline, Philadelphia, PA, USA) were administered to participants with FEV_1_:FVC<0.7 and spirometry was repeated after 10 minutes.

An external spirometry review committee (Health Research Associates, Concord, USA) reviewed all tests for ATS acceptability and repeatability. Results were interpreted using National Health and Nutrition Examination Survey III (NHANES III) prediction equations with African American correction factors [15] and standard interpretation criteria [16]. NHANES III prediction equations were chosen based on similarities with east African prediction equations [17]. Tests were considered reproducible if the FEV_1_ and FVC from the two best trials were within 200 mL (mL) of one another. Tests with only two trials were included if they met repeatability criteria. COPD was defined as a post-bronchodilator FEV_1_:FVC less than the fifth percentile of the predicted values (lower limit normal; LLN), which were defined using NHANES III prediction equations.

All study procedures were conducted in accordance with the guidelines of the Declaration of Helsinki and were approved by the Mbarara University of Science and Technology and Partners Healthcare human studies ethics committees, the Uganda National Council for Science and Technology, and the Research Secretariat in the Office of the President (Uganda). All study participants provided written informed consent.

### Statistical analysis

To evaluate for selection bias, cohort demographics were compared between 1) health screenings attendees and non-attendees, 2) attendees who completed and declined spirometry, and 3) attendees with ATS acceptable and unacceptable spirometry.

Inverse probability of treatment weights (IPTW) were used to estimate population prevalence, whereby we first estimated the propensity to participate in the health fair using a logistic regression model consisting of 15 variables collected during the census that were expected to correlate with health fair attendance: age, sex, education level, marital status, village of residence, household asset wealth, distance from the health fair, difference between household altitude and health fair altitude, self-reported overall health, self-reported HIV status, heavy alcohol use, social network size, index of social participation, water insecurity and food insecurity. We defined household asset wealth using the method developed by Filmer and Pritchett [18], social network size as the total number of unique social network ties elicited in response to a series of culturally-adapted social network name generator questions (which can be informally understood as a participant’s total number of “friends”) [19], social participation as the number of different community groups in which the participant reported significant participation in the previous two months [20], and water and/or food insecurity as having limited or uncertain availability of safe water and/or nutritious food or lacking the ability to acquire safe water and/or nutritious food in socially acceptable ways [10,21]. We using this model to calculate stabilized IPTWs using methods developed by Hernan et al [22].

Nyakabare Parish-representative estimates of COPD and respiratory symptom prevalence were generated by incorporating IPTWs using the svy command in Stata (StataCorp LLC, College Station, TX, USA), and sensitivity to extreme IPTW weights was assessed by excluding those with weights at the 99th/1st, 95th/5th and 90th/10th percentiles. Our primary outcome of interest was lung function, as measured by FEV_1_ and FEV_1_: FVC. Our secondary outcome of interest was chronic respiratory symptoms, defined as either chronic cough or chronic dyspnea. Explanatory covariates of interest were chosen based on the scientific plausibility of their association with either lung function or respiratory symptoms and included age, sex, smoking history, education level, household asset index (as a measure of socioeconomic status [18]), body mass index and HIV serostatus. The joint statistical significance of regression coefficients on categorical variables (eg, household asset index quintiles) was evaluated using a Wald-type F-test.

Multivariable log binomial regression models were fit to estimate associations between chronic respiratory symptoms and covariates of interest, and multivariable linear regression models were fit to estimate associations between lung function and covariates of interest. Participants with missing data for any model covariates were excluded from analyses. Sensitivity analyses were conducted in which the study population was restricted to a) participants at least 40 years of age, and b) those for whom the two best spirometry trials were within 150 mL of one another. Data were analyzed using Stata 13 (StataCorp LLC, College Station, TX, USA).

## RESULTS

**Figure 1:**
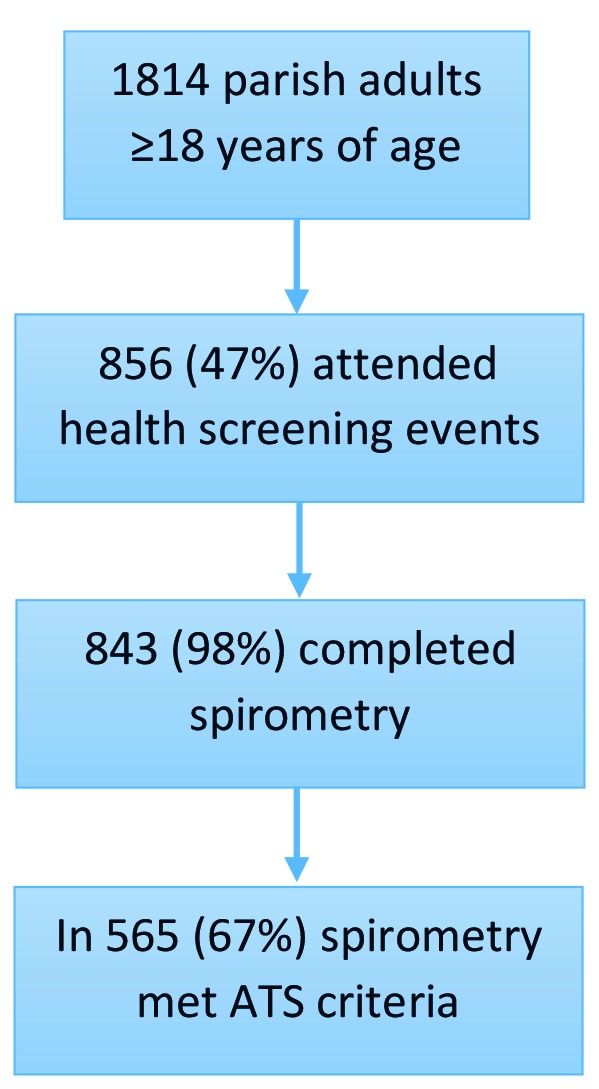
Flow diagram of participant selection. Flow diagram describing the number of eligible participants in the target region, how many eligible participants sought screening at the health fair, and how many of those who sought screening completed respiratory questionnaires and American Thoracic Society (ATS)-acceptable pulmonary function testing. Those who completed respiratory questionnaires and ATS-acceptable pulmonary function testing were used to calculate respiratory symptom burden and COPD prevalence estimates, respectively.

Out of 1814 enumerated Nyakabare Parish residents, 47% (n = 856) attended the health screenings, most of whom (98%, 843/856) completed medical questionnaires and spirometry (**Figure 1**). Most spirometry (67%, n = 565) met interpretation criteria. Compared with those who did not attend the health fairs, participants who attended were older, more commonly women, less educated, and reported lower subjective health (*P* < 0.001 for all comparisons, Supplementary Table S1 in the Online Supplementary Document). There were no statistically nor substantively significant differences between those who completed spirometry as compared to those who declined spirometry, or between those whose spirometry met ATS standards as compared to those whose spirometry was unacceptable (Tables S2 and S3 in **Online Supplementary Document**).

Using IPTWs to model population-level data, the population was half women (52%), with a mean age of 39 ± 17 years, most of whom (75%) had never smoked (**Table 1**). Over half (59%) had completed at most a primary school education, 62% were subsistence farmers, HIV prevalence was 5%, and few had been diagnosed with COPD (1%) or asthma (1%). These estimates are similar to general Ugandan population demographics [9,23]. Chronic respiratory symptoms were reported by 30% of the population, which was unchanged when excluding participants with bronchodilator-associated reversibility on spirometry (**Table 2**). Among those who reported a cough, most (70%) reported regular sputum production.

**Table 1:** Cohort characteristics of participants who completed ATS-acceptable spirometry and population characteristics

Characteristic	Unweighted study sample (n = 565)*	Population weighted estimates
**Age, years (mean, standard deviation)**	43 ± 17	39 ± 17
**Women**	62% (n = 348)	52%
**Smoking history:**		
Never smoker	71% (n = 398)	75%
Former smoker	19% (n = 107)	15%
Current smoker	10% (n = 57)	10%
Years smoked†	20 (±17)	20 (±16)
Farmer	71% (n = 399)	62%
**Body mass index (kg/m^3^):**		
Underweight (<18.5)	5% (n = 26)	4%
Normal (18.5-25)	54% (n = 305)	61%
Overweight (25.1-30)	26% (n = 147)	22%
Obese (≥30)	15% (n = 87)	12%
**Education:**		
None	17% (n = 95)	13%
Some primary school	32% (n = 182)	24%
Completed primary school	25% (n = 142)	22%
At least some secondary school	26% (n = 146)	40%
**Medical comorbidities:‡**		
COPD	1% (n = 4)	1%
Asthma	2% (n = 9)	1%
HIV	5% (n = 25)	5%

ATS – American Thoracic Society, COPD – chronic obstructive pulmonary disease, HIV – human immunodeficiency virus

*Missing data were present for the following covariates – Smoking history: 1% (n = 3); Farmer: 6% (n = 33); HIV: 12% (n = 65); COPD: 2% (n = 12); Asthma: 1% (n = 5).

†Current or former smokers only.

‡COPD and Asthma were self-reported. HIV serostatus was confirmed with antibody testing.

**Table 2:** Mean lung function (% predicted), COPD prevalence and cough/dyspnea prevalence, under progressive truncation of overall weights*

Outcome	Total population (n = 565)	Weighted population estimate without trimming (95% CI)		Trimmed^†^ weight population estimates (95% CI)		
		**99^th^, 1^st^ percentile**		**95^th^, 5^th^ percentile**	**90^th^, 10^th^ percentile**
FEV_1_ (% predicted)	99.1 (19.4)	100.2 (98.1,102.3)	99.6 (97.9, 101.3)		99.3 (97.7, 100.9)	99.2 (97.6, 100.7)
FVC (% predicted)	100.9 (17.9)	101.7 (100.0, 103.5)	101.2 (99.7, 102.8)		101.0 (99.5, 102.4)	100.9 (99.4, 102.3)
FEV_1_:FVC (% predicted)	97.9 (7.3)	98.7 (97.6, 99.7)	98.5 (97.7, 99.2)		98.4 (97.6, 99.0)	98.2 (97.6, 98.9)
COPD prevalence	2.3	1.6 (0.9, 2.9)	1.7 (1.0, 3.1)		1.9 (1.0, 3.3)	2.0 (1.1, 3.5)
-Age ≥40‡	3.3	2.9 (1.4, 5.7)	2.9 (1.4, 5.7)		3.0 (1.5, 5.9)	3.1 (1.5, 6.1)
-Best values ≤150 mL§	2.5	2.0 (0.9, 4.3)	2.2 (1.0, 4.6)		2.3 (1.1, 4.8)	2.3 (1.2, 4.4)
Cough/dyspnea prevalence	35.0	30.3 (25.1, 36.0)	32.2 (27.6, 37.1)		33.1 (28.8, 37.7)	33.4 (29.3, 37.8)
-Excluding BD reversibility	35.0	30.1 (24.9, 35.9)	32.0 (27.4, 37.0)		32.9 (28.6, 37.5)	33.2 (29.2, 37.6)

CI – confidence interval, FEV_1_ – Forced expiratory volume in one second, FVC – Forced vital capacity, % – predicted: percent predicted using National Health and Nutrition Examination Survey III prediction equations, COPD – chronic obstructive pulmonary disease, BD – bronchodilator

*Mean (standard deviation) or weighted population mean (95% CI) unless otherwise noted.

†Trimmed weights replace values above and below the respective percentiles (eg, 99^th^/1^st^ percentile) with the values at that percentile to ensure estimates are not excessively sensitive to extreme values

‡n = 274.

§n = 397.

Mean percent predicted lung function was normal. Population prevalence of COPD was 2%, which was unchanged when defining COPD as FEV_1_:FVC<0.7 or restricting acceptable spirometry to that in which the best trials were within 150mL of one another (**Table 2**). Prevalence increased minimally to 3% when limiting the cohort to participants at least 40 years old, and estimates were similar when utilizing trimmed weights. COPD prevalence was higher among older individuals (*P* = 0.005) and ever-smokers (*P* = 0.002) (Table S4 in **Online Supplementary Document**).

In multivariable log binomial regression models adjusted for potential confounders, the prevalence of chronic respiratory symptoms was 71% higher among women (*P* = 0.006) and 50% higher among current or former smokers (*P* = 0.04) (Table S5 in the Online Supplementary Document). In multivariable linear regression models adjusted for potential confounders, FEV_1_ and FEV_1_:FVC were lower among women and ever-smokers (**Table 3**). Conversely, FEV_1_ was higher among older individuals. Effect estimates were similar in sensitivity analyses (Tables S6-S10 in **Online Supplementary Document**).

**Table 3:** Population-weighted unadjusted and adjusted correlates of lung function*

Characteristic	FEV_1_ (% predicted)				FEV_1_:FVC (% predicted)			
**Unadjusted**		**Adjusted**		**Unadjusted**		**Adjusted**	
**% Δ**	**95% CI**	**% Δ**	**95% CI**	**% Δ**	**95% CI**	**% Δ**	**95% CI**
Age, per year	0.08	-0.01, 0.17	0.15	0.03, 0.26	0.01	-0.02, 0.05	0.04	-0.01, 0.08
Female sex	-5.15	-8.04, -2.26	-6.02	-9.31, -2.73	-4.36	-5.51, -3.20	-4.36	-5.66, -3.06
Smoking	-0.18	-3.55, 3.18	-5.91	-10.41, -1.42	-0.39	-1.79, 1.01	-2.31	-4.09, -0.54
Cough or dyspnea	-4.97	-8.15, -1.79	-2.53	-5.98, 0.92	-1.73	-3.06, -0.41	-0.63	-1.99, 0.73
Asset ownership index:								
-Poorest	-2.95	-7.51, 1.60	-0.58	-5.52, 4.35	-1.87	-3.75, 0.01	-1.66	-3.62, 0.29
-Poorer	0.82	-3.90, 5.53	1.50	-3.59, 6.60	-0.32	-2.26, 1.63	-0.42	-2.43, 1.59
-Middle	4.10	-0.12, 8.30	4.62	0.14, 9.10	1.45	-0.29, 3.18	0.29	-1.49, 2.06
-Richer	1.03	-3.53, 5.58	0.87	-4.02, 5.76	1.65	-0.23, 3.53	0.13	-1.80, 2.06
HIV	-5.18	-12.09, 1.73	-5.71	-12.76, 1.33	-1.32	-4.14, 1.49	-1.07	-3.85, 1.72

FEV_1_ – Forced expiratory volume in one second, FVC – Forced vital capacity, % predicted – percent predicted using National Health and Nutrition Examination Survey III prediction equations, %Δ – percent change, CI – confidence interval, HIV – human immunodeficiency virus

*Missing data were present for the following model covariates: smoking history: 1% (n = 3); cough or dyspnea: 1% (n = 7); asset ownership index: 1% (n = 5); HIV: 12% (n = 65). Reference categories for comparisons: Smoking – lifelong non-smoker; Asset ownership index – Richest quintile.

## DISCUSSION

In rural southwestern Uganda, we estimated a lower than expected population prevalence of COPD despite a high respiratory symptom burden. Our results add to a growing body of literature investigating the burden of COPD-related morbidity and mortality across SSA that may challenge widely held assumptions that COPD is among the top chronic health conditions in the region.

This study adds to a handful of population-based studies in SSA demonstrating a low prevalence of COPD (**Table 4**) [7,25-28]. Our COPD prevalence estimates are similar to those described by Siddharthan and Grigsby et al., who found that COPD population prevalence in Uganda ranged from 1.7% to 7.4% among urban and rural Ugandan cohorts, respectively, despite nearly ubiquitous biomass exposure [7]. There are several potential explanations for the lower-than-expected COPD prevalence in our cohorts. First, biomass smoke exposure is predicted to be the leading cause of COPD in SSA [38], yet a recent multi-site study of 18 554 adults across 25 different sites found no relationship between biomass exposure and COPD [39]. Biomass smoke-associated COPD is characterized by more small airways disease, less emphysema and a slower FEV_1_ decline over time as compared to cigarette smoke-associated COPD [40,41]. Thus, studies utilizing imaging to evaluate structural lung abnormalities may be necessary to better understand the true burden of biomass-associated lung disease.

**Table 4:** COPD prevalence estimates in sub-Saharan Africa

First author	Publication year	Country	Setting	Sample size	Mean age (years)	COPD prevalence
Siddharthan, Grigsby [7]	2018	Uganda	Urban	596	44.2	1.7%
Siddharthan, Grigsby [7]	2018	Uganda	Rural	721	49.1	7.4%
Magitta [24]	2018	Tanzania	Rural	496	51.8	11.1%
Pefura-Yone [25]	2016	Cameroon	Urban	1,287	34.4	2.4%
Meghji [26]	2016	Malawi	Urban	748	41.9	4.2%
Obaseki [27]	2016	Nigeria	Urban	875	Not provided	7.7%
van Gemert [6]	2015	Uganda	Rural	588	45	16.2%
Musafiri [28]	2011	Rwanda	Urban/Rural	1,824	38.3	4.5%
Fullerton [29]	2011	Malawi	Urban/Rural	332	46	13.6%
Khelafi [30]	2011	Algeria	Urban/Rural	1800	47.5	4.9%
Martins [31]	2009	Cape Verde	Urban/Rural	274	41.7	8.4%
Girdler-Brown [32]	2008	South Africa	Prior gold miners	624	49.4	13.4%
Buist [33]	2007	South Africa	Urban	847	53.6	23.8%
Gathuru [34]	2002	Nigeria	Urban	270	47.6	9.3%
Oleru, Onyekwere [35]	1992	Nigeria	Urban shoe factory	134	33.1	6.8%
Myers, Cornell [36]	1989	South Africa	Urban brick workers	268	29.6	4.1%
Wicht [37]	1977	South Africa	Urban	509	(20-79)	9.3%

COPD – Chronic obstructive pulmonary disease

Several additional explanations may also contribute to the lower-than-expected COPD population prevalence. Lower FEV_1_ is associated with increased all-cause and respiratory mortality [42], so survivorship bias may have caused us to underestimate the prevalence of COPD in the general population. Consistent with this theory, we found that older age was paradoxically associated with better lung function. Indeed, while still lower than expected, the unadjusted COPD prevalence was highest within the oldest quartile of our study participants. Additionally, smoking is less common in SSA compared to high-income countries, and for those who do smoke, the overall tobacco exposure is much lower [43]. Thus, tobacco exposure in this and other SSA cohorts may not be high enough to cause the burden of COPD seen in populations with higher tobacco exposure.

Importantly, several studies in SSA have measured higher COPD prevalence compared to our results. Investigators in the Masindi district of western Uganda diagnosed COPD in 16% of 588 adults [6]. Our cohorts are quite similar in terms of age, sex, educational attainment, occupation, and HIV prevalence. Though we did not collect biomass exposure data in this cohort, other work in the Mbarara District of southwestern Uganda confirms ubiquitous biomass exposure [44,45] similar to that reported in the Masindi cohort. The differences in our COPD prevalence estimates may be related to variations in tobacco exposure, urban vs rural environment, and tribal ancestry. The Masindi District is one of the main tobacco growing regions in Uganda [46], and more of the Masindi cohort participants are current or former smokers as compared to our cohort (36% vs 29%) [47]. Additionally, 17% of the Masindi cohort lives in urban regions, where lung disease risks such as air pollution are more prevalent, while our cohort resides entirely in a rural setting. Lastly, tribal ancestry differs between western and southwestern Uganda. The major tribes of the Masindi district in western Uganda are the Banyoro and Lugbara, while southwestern Uganda is populated mainly by people of Banyankole descent [48], and differences in ethnicity are associated with variations in lung function [49].

The highest COPD population prevalence estimate in SSA was measured in urban South Africa, where 24% of cohort participants were diagnosed with COPD [33]. While tobacco exposure was also higher in the South African cohort, tuberculosis incidence in South Africa is among the highest in the world, and post-tuberculosis obstructive lung disease is emerging as a commonly-described post-infectious complication [50]. Our COPD population prevalence estimates are also lower than the prevalence estimates from two recent meta-analyses of spirometry-based studies in SSA. Based on data from seven African studies from 1999 to 2010, Adeloye and colleagues estimate that 10.6-13.4% of the aggregate study population has COPD [3,51]. Heterogeneity in study methods and cohort characteristics among the analyzed studies, including several that estimate cohort prevalence rather than population prevalence, may explain the differences compared to our prevalence estimates.

We found that lung function was lower among women and ever-smokers, consistent with published literature [52,53]. At similar levels of tobacco exposure, female smokers have higher COPD risk and more accelerated lung function decline compared to male smokers,[53] which may be related to sex-based risk differences [54]. In Uganda and elsewhere in SSA, women are also disproportionately exposed to biomass smoke due to sex-based meal preparation roles [55]. As noted above, associations between biomass smoke exposure and impaired lung function have been found by some [7] but not all [39] investigators, and thus require further study in the context of our findings. We found no associations between HIV serostatus and lung function, consistent with some [26] but not all [56] studies in SSA. Chronic HIV infection increases risk for COPD due to virus-associated chronic inflammation and immune activation [57], which takes many years to develop. The HIV-infected individuals in our cohort were at varying stages of disease, which may explain the lack of association.

### Respiratory symptom prevalence

Ours is the first study to estimate the population burden of chronic respiratory symptoms in rural Uganda. In contrast to COPD prevalence, we found an unexpectedly high prevalence of chronic respiratory symptoms in this cohort. There are several hypotheses that may explain the discrepancies in these prevalence estimates. First, respiratory symptoms are often caused by extra-pulmonary disease. For example, the differential diagnosis of chronic cough includes post-nasal drip and gastroesophageal reflux, and allergic upper airway irritation can cause cough and sputum production [58]. Cardiac dysfunction can also cause cough and dyspnea [59,60]. Though cardiac arrhythmias were uncommon in this cohort [61], we did not evaluate cardiac function per se. Second, respiratory symptoms may be due to non-obstructive lung disease. For instance, though we did not estimate the prevalence of restrictive lung disease in this cohort, investigators in Malawi found that 38.6% of 749 urban adults had restrictive physiology while only 3.6% had moderate to severe airways obstruction [26]. Spirometry more readily identifies airways abnormalities compared with lung parenchymal abnormalities. Pulmonary tuberculosis and other lower respiratory tract infections are prevalent in sub-Saharan Africa [62,63] and can cause subsequent interstitial lung abnormalities [64,65]. Air pollution exposure has also been associated with interstitial lung abnormalities in adults and children [66,67]. It is possible that the participants in our cohort with respiratory symptoms and normal spirometry may have undiagnosed interstitial lung disease that would be more accurately identified with chest computed tomography. To investigate these hypotheses, we are now planning to collect high-resolution chest computed tomography imaging in a subset of study participants to further characterize the burden of chronic respiratory disease in this cohort.

The prevalence of chronic cough in the study population was over 10 times higher than estimates from a meta-analysis of African studies [68]. Although a handful of prior studies have reported similar respiratory symptom prevalence estimates in Uganda, Nigeria, South Africa and Malawi [69-72], prevalence estimates in similarly rural locations are often much lower. For example, participants in a longitudinal cohort of HIV-infected adults on antiretroviral therapy in rural Uganda reported chronic cough at 10% of study visits [45]. Similarly, the prevalence of cough was 7.5% among a community-based cohort of Malawian adults [26], 1.2% among rural Gambian adults [73], and 5% among a cohort of Rwandan adults [28]; although the definitions of cough varied among these studies.

The high respiratory symptom prevalence in our rural cohort may be related to air pollution sources such as biomass fuels. Consistent with published literature, respiratory symptom prevalence was significantly higher among women, who may experience higher exposure to inhaled irritants from biomass-related household air pollution [55] or have a more sensitive cough reflex when compared to men [74]. As previously described, women may also be more susceptible to the respiratory effects of inhalation-based exposures [53,54]. Compared to SSA cohorts reporting lower respiratory symptom prevalence, home biomass exposure in our cohort was ubiquitous.

The main strength of this analysis is the use of a large, population-based cohort with complete demographic data on those who did not complete study procedures, which allowed us to calculate population disease prevalence estimates. This study also has several limitations. First, only half of the study cohort attended the health screenings, which may have introduced selection bias if those with previously diagnosed COPD and/or those in poor health were less likely to attend. However, spirometry availability in the region is minimal and travel was facilitated for those in poor health, which limits the influence of potential selection bias on our prevalence estimates. Second, those who completed health screenings were older and more likely to feel unhealthy, which may have caused us to overestimate COPD prevalence. However, the measured COPD prevalence was lower than expected, so any potential upward bias that may have resulted would only further accentuate our conclusion that COPD is uncommon. The IPTW estimates also leveraged data from the entire cohort to produce population-representative estimates based on data obtained from those who did not attend the health screenings, although population-weighted prevalence estimates may not fully represent the true population prevalence if prevalence varies by some unmeasured confounder. Third, about one third of spirometry results did not meet interpretation criteria, but there were no statistically significant differences between participants with and without acceptable spirometry, so this is unlikely to have systematically biased our results. Additionally, bronchodilator was only administered to those with FEV1/FVC<0.7 rather than those with FEV1/FVC<LLN due to automated spirometer algorithms, which may have caused us to underestimate COPD prevalence in this younger cohort. However, COPD prevalence estimates were similar when restricting the cohort to those over 40 years old. Also, our study participants were drawn from one region in rural southwestern Uganda, and COPD prevalence may vary by region due to differences in risk factors or health-related behaviors. While our estimates vary from those described in rural western Uganda, they are similar to those described in rural central Uganda, which further emphasizes the importance of measuring disease prevalence in various locations. Lastly, no data were collected on biomass fuel exposure, but biomass use in this region is ubiquitous[45] and thus unlikely to have biased our results.

## CONCLUSIONS

In conclusion, COPD prevalence among adults attending health screenings in rural southwestern Uganda is low despite prevalent respiratory symptoms. If the results of our work are corroborated, further work is needed to evaluate whether the underlying etiology of the high respiratory symptom burden in rural southwestern Uganda represents underlying structural lung disease not identified by screening spirometry.

## Additional material

Online Supplementary Document
