# Supplementary material for: Prevalence and correlates of chronic obstructive pulmonary disease and chronic respiratory symptoms in rural southwestern Uganda: a cross-sectional, population-based study
Source: J Glob Health. 2019 Jun 15;9(1):010434. doi: 10.7189/jogh.09.010434 (PMC6571107; doi:10.7189/jogh.09.010434)
Supplement: Online Supplementary Document [file jogh-09-010434-s001.pdf]

J Glob Health 2019, 9: 010434

Data Supplement

Title: Prevalence and correlates of chronic obstructive pulmonary disease and chronic respiratory symptoms in rural Uganda: a cross-sectional, population-based study

Authors: Crystal M. North,[1-3] Bernard Kakuhikire,[4] Dagmar Vořechovská,[1] Simone Hausammann-Kigozi,[4] Amy Q. McDonough,[1] Jordan Downey,[5] David C. Christiani,[1-3] Alexander C. Tsai,[1,2,4] Mark J. Siedner[1,2,4]

Institutional Affiliations: 1) Massachusetts General Hospital, Boston, Massachusetts, USA; 2) Harvard Medical School, Boston, Massachusetts, USA; 3) Harvard T.H. Chan School of Public Health, Boston, Massachusetts, USA 4) Mbarara University of Science and Technology, Mbarara, Uganda; 5) Last Mile Health, Cambridge, Massachusetts, USA

Figure S1. Health screening locations in Nyakabare Parish, southwestern Uganda

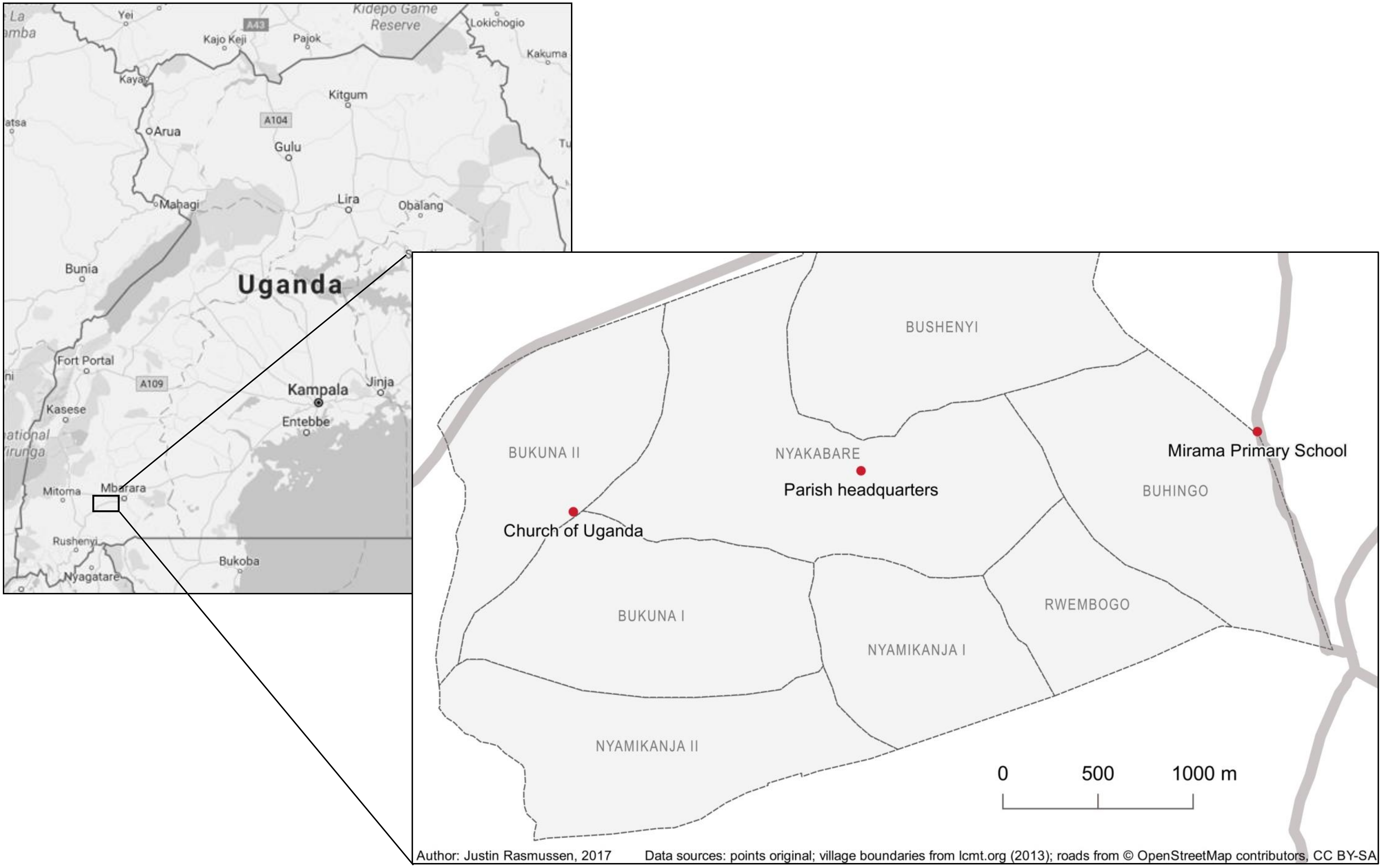

Figure S1 Legend

Location of Nyakabare Parish within a larger map of Uganda, where the red dots indicate individual health screening event locations superimposed on a map delineating the geography of Nyakabare parish and its constituent cells (akin to neighborhoods). Thick gray lines indicate nearby roads, thin dashed gray lines indicate parish and cell boundaries. Health screenings were held at the Church of Uganda on June 25th and 26th, 2015; at the Mirama Primary School on June 27th, 2015; and at Parish headquarters on June 28th and 29th, 2015. The map of Uganda is courtesy of Google Maps [December 18, 2017], available at: https://www.google.com/maps/place/Uganda and the map of Nyakabare Parish and health screening event locations is courtesy of Mr. Justin Rasmussen, 2017.

Figure S2. Respiratory Questionnaire

Has a physician, other health care worker, or traditional healer ever told you that you have chronic obstructive pulmonary disease (COPD)?

- In what year or at what age were you diagnosed with chronic obstructive pulmonary disease (COPD)?
- Have you been prescribed medicines for your chronic obstructive pulmonary disease (COPD)?
- Are you currently taking medicines for your chronic obstructive pulmonary disease (COPD)?

Has a physician, other health care worker, or traditional healer ever told you that you have asthma?

- In what year or at what age were you diagnosed with asthma?
- Have you been prescribed medicines for your asthma?
- Are you currently taking medicines for your asthma?

Do you have a cough on most days?

Do you cough up sputum on most days?

Do you have difficulty in breathing on most days?

In the past, have you smoked tobacco such as cigarettes or pipes on a daily basis, less than daily, or not at all?

Do you currently smoke tobacco, such as cigarettes or pipes?

What year (or age) did you first begin smoking?

What year (or age) did you quit smoking?

Table S1: Cohort characteristics of participants who attended the health screenings compared to those who did not attend

Characteristic	Attendees (n = 856)	Non-attendees (n = 955)	*P* value
Age	42 (± 18)	34 (± 15)	<0.001
Female sex	534 (62)	464 (49)	<0.001
Education level			<0.001
None	158 (18)	111 (12)	
Some primary	293 (34)	220 (23)	
Completed primary	201 (23)	198 (21)	
At least some secondary	204 (24)	423 (44)	
Self-reported health			<0.001
Very bad	12 (1)	7 (1)	
Bad	228 (27)	125 (13)	
Good	508 (59)	677 (71)	
Very good	108 (13)	143 (15)	

Table S1 Legend

n (%) or mean (±SD)

Table S2: Comparisons between demographic characteristics of health screening attendees who completed spirometry compared with those who declined spirometry

Characteristic	Completed Spirometry (n = 843)	Declined Spirometry (n = 14)	*P* value
Age	43 (± 18)	46 (± 17)	0.53
Female sex	525 (62)	10 (71)	0.59
Smoking			0.25
Never smoker	577 (69)	12 (86)	
Current/former smoker	259 (31)	2 (14)	
HIV	39 (5)	1 (13)	0.36
Farmer	594 (74)	12 (86)	0.54
Education level			0.06
None	157 (19)	1 (7)	
Some primary	283 (34)	10 (71)	
Completed primary	201 (24)	1 (7)	
At least some secondary	202 (24)	2 (14)	
Asset ownership index			0.58
Poorest	167 (20)	5 (38)	
Poorer	169 (20)	3 (23)	
Middle	172 (21)	2 (15)	
Richer	167 (20)	2 (15)	
Richest	157 (19)	1 (8)	
Respiratory symptoms	310 (37)	4 (29)	0.59
History of COPD	7 (1)	0 (0)	1.0
History of Asthma	11 (1)	0 (0)	1.0
Poor self-reported health	5 (36)	235 (28)	0.52

Table S2 Legend

HIV: Human Immunodeficiency Virus

n (%) or mean (±SD)

Table S3: Comparisons between demographic characteristics of health fair attendees who completed ATS-acceptable spirometry compared with those who were unable to complete acceptable spirometry

**Characteristic**	**ATS-acceptable spirometry (n = 565)**	**ATS-unacceptable spirometry (n = 278)**	***P* value**
Age	43 (± 20)	43 (± 17)	0.83
Female sex	348 (62)	177 (64)	0.56
Smoking			0.11
Never smoker	398 (71)	179 (65)	
Current/former smoker	164 (29)	95 (35)	
HIV	25 (5)	14 (6)	0.64
Farmer	399 (75)	195 (73)	
Education level			0.053
None	95 (17)	62 (22)	
Some primary	182 (32)	101 (36)	
Completed primary	142 (25)	59 (21)	
At least some secondary	146 (26)	56 (20)	
Household asset index			0.07
Poorest	103 (18)	64 (24)	
Poorer	104 (19)	65 (24)	
Middle	119 (21)	53 (19)	
Richer	121 (22)	46 (17)	
Richest	113 (20)	44 (16)	
Respiratory symptoms	195 (35)	115 (42)	0.06
History of COPD	4 (1)	3 (1)	0.69
History of Asthma	9 (2)	2 (1)	0.52
Poor self-reported health	149 (26)	86 (31)	0.16

Table S3 Legend

ATS: American Thoracic Society

Table S4. COPD prevalence among selected sub-groups

Characteristic	Unweighted Study Sample (n = 565)	*p* value	Population Weighted Estimates	*P* value
Prevalence	2% (n=13)		1.6%	
Age		0.03		0.005
18–29	0% (n=0)		0%	
30–39	0% (n=0)		0.2%	
40–53	3% (n=4)		0.9%	
54–93	5% (n=7)		1.6%	
Sex		0.09		0.29
Male	4% (n=8)		1%	
Female	1% (n=5)		1.6%	
Smoking history		0.03		0.002
Ever smoker	5% (n=8)		1.0%	
Never smoker	1% (n=5)		0.6%	
Asset index		0.38		0.51
Poorest	5% (n=5)		0.5%	
Poorer	3% (n=3)		0.5%	
Middle	2% (n=2)		0.2%	
Richer	1% (n=1)		0.2%	
Richest	2% (n=2)		0.3%	
Education		0.06		0.02
None	5% (n=5)		0.6%	
At least some primary school	1% (n=4)		0.4%	
At least some secondary school	3% (n=4)		0.6%	
Occupation		0.75		0.67
Farmer	2% (n=9)		1.2%	

Other professions	3% (n=4)		0.6%	
HIV serostatus		1.0		0.53
HIV positive	0% (n=0)		0%	
HIV negative	2% (n=10)		1.3%	
Respiratory symptoms		0.15		0.09
Cough or dyspnea	4% (n=7)		0.9%	
None	2% (n=6)		0.8%	

Table S4 Legend

COPD: Chronic obstructive pulmonary disease; HIV: Human immunodeficiency virus

Table S5. Population-weighted prevalence ratios for predictors of self-reported cough or dyspnea

	**Unweighted Study Sample (n=565)**	**Population-Weighted Estimates**	
**Characteristic**	*Unadjusted PR (95% CI)*	*Unadjusted PR (95% CI)*	*Adjusted PR (95% CI)*
Age, per year	1.00 (0.99, 1.01)	1.01 (1.00, 1.02)	1.00 (0.99, 1.01)
Female gender	1.64 (1.13, 2.36)	1.76 (1.18, 2.62)	1.71 (1.17, 2.51)
Smoking	1.50 (1.03, 2.19)	1.47 (1.06, 2.03)	1.50 (1.02, 2.18)
Education			
Some primary school	0.80 (0.48, 1.32)	0.85 (0.62, 1.16)	
Completed primary school	0.49 (0.28, 0.84)	0.63 (0.43, 0.94)	
Some secondary school	0.36 (0.21, 0.63)	0.34 (0.20, 0.58)	
Asset index			
Poorest	1.73 (0.99, 3.01)	1.61 (1.00, 2.59)	1.26 (0.78, 2.03)
Poorer	1.08 (0.61, 1.88)	1.27 (0.77, 2.10)	1.14 (0.69, 1.88)
Middle	0.85 (0.49, 1.49)	0.69 (0.36, 1.32)	0.74 (0.42, 1.33)
Richer	0.84 (0.49, 1.47)	1.05 (0.61, 1.82)	0.86 (0.51, 1.45)
BMI			
Underweight (<18.5)	1.92 (0.86, 4.32)	1.66 (0.93, 2.97)	1.34 (0.84, 2.13)
Overweight (25–29.9)	1.41 (0.93, 2.14)	1.39 (0.95, 2.04)	1.06 (0.70, 1.61)
Obese (≥30)	1.50 (0.91, 2.47)	1.56 (1.00, 2.41)	1.37 (0.86, 2.17)
HIV	0.61 (0.24, 1.57)	0.85 (0.37, 1.95)	0.83 (0.36, 1.92)

Table S5 Legend

PR: Prevalence Ratio; CI: Confidence interval; BMI: Body mass index; HIV: Human immunodeficiency virus

Reference categories for comparisons: Smoking - lifelong non-smoker; Education - none; Asset Index - Richest quintile; BMI - normal (18.5 - 24.9)

Table S6. Population-weighted correlates of lung function; sensitivity analysis restricted to participants at least 40 years of age (n = 240)

Characteristic	FEV_1 (% predicted)				FEV_1:FVC (% predicted)			
	Unadjusted		Adjusted		Unadjusted		Adjusted	
	% Δ	*95% CI*	*% Δ*	*95% CI*	*% Δ*	*95% CI*	*% Δ*	*95% CI*
Age, per year	0.3	0.1, 0.5	0.3	0.02, 0.5	0.01	-0.1, 0.1	0.03	-0.06, 0.1
Female sex	2.1	-3.0, 7.1	1.1	-4.8, 7.1	-1.4	-3.3, 0.5	-2.5	-4.8, -0.3
Smoking	-1.4	-6.4, 3.7	-6.0	-12.3, 0.2	-2.0	-3.8, -0.1	-2.6	-5.0, -0.2
Cough or dyspnea	-5.8	-11.2, -0.5	-4.0	-9.8, 1.9	-1.4	-3.4, 0.6	-0.1	-2.4, 2.1
Asset ownership index								
Poorest	-5.0	-12.7, 2.8	-1.2	-10.1, 7.7	-3.9	-6.8, -1.0	-2.0	-5.4, 1.4
Poorer	2.2	-5.9, 10.3	4.3	-5.0, 13.7	-3.7	-6.7, -0.7	-1.8	-5.4, 1.8
Middle	5.0	-2.5, 12.4	9.2	1.1, 17.3	-1.1	-3.8, 1.7	-0.1	-3.2, 3.1
Richer	1.4	-6.2, 9.0	2.5	-5.9, 10.9	-0.4	-3.2, 2.4	-0.7	-4.0, 2.5
HIV	-6.0	-15.5, 3.5	-8.5	-18.4, 1.4	0.7	-2.9, 4.3	0.3	-3.5, 4.1

Table S6 Legend

FEV_1: Forced expiratory volume in one second; FVC: Forced vital capacity; CI: Confidence interval; % predicted: percent predicted; % Δ: percent change

Reference categories for comparison: Smoking - lifelong non-smoker; Asset Index - Richest quintile

Table S7. Population prevalence of COPD among selected sub-groups; sensitivity analysis restricted to participants at least 40 years of age

Characteristic	Unweighted Study Sample (n = 290)	*p* value	Population Weighted Estimates	*P* value
Prevalence	3% (n=9)		2.7%	
Age		1.0		0.67
40–53	3% (n=4)		1.2%	
54–93	3% (n=5)		1.5%	
Gender		0.16		0.06
Male	5% (n=6)		2.0%	
Female	2% (n=3)		0.7%	
Smoking history		0.33		0.37
Ever smoker	4% (n=6)		1.8%	
Never smoker	2% (n=3)		0.9%	
Asset index		0.48		0.25
Poorest	6% (n=3)		0.7%	
Poorer	6% (n=3)		1.1%	
Middle	2% (n=1)		0.2%	
Richer	1% (n=1)		0.4%	
Richest	2% (n=1)		0.2%	
Education		0.14		0.13
None	5% (n=4)		1.1%	
At least some 1˚ school	1% (n=2)		0.5%	

At least some 2˚ school	5% (n=3)		1.2%	
Occupation		0.07		0.15
Farmer	2% (n=5)		1.6%	
Other professions	7% (n=4)		1.3%	
HIV serostatus		1.0		0.49
HIV positive	0% (n=0)		0%	
HIV negative	3% (n=6)		2.0%	
Respiratory symptoms		0.29		0.10
Cough or dyspnea	5% (n=5)		1.7%	
None	2% (n=4)		1.1%	

Table S7 Legend

COPD: Chronic obstructive pulmonary disease; HIV: Human immunodeficiency virus; 1˚: Primary; 2˚: Secondary

Table S8. Population-weighted correlates of lung function; sensitivity analysis restricted to participants whose best spirometry trials were within 150mL (n = 349)

Characteristic	FEV_1 (% predicted)				FEV_1:FVC (% predicted)			
	Unadjusted		Adjusted		Unadjusted		Adjusted	
	% Δ	*95% CI*	*% Δ*	*95% CI*	*% Δ*	*95% CI*	*% Δ*	*95% CI*
Age, per year	0.1	0.02, 0.2	0.2	0.04, 0.3	-0.01	-0.05, 0.04	0.02	-0.03, 0.1
Female sex	-6.3	-9.8, -2.9	-6.1	-10.1, -2.1	-5.0	-6.3, -3.7	-4.2	-5.6, -2.7
Smoking	3.3	-0.7, 7.3	-1.0	-6.4, 4.3	-0.2	-1.8, 1.4	-1.3	-3.3, 0.6
Cough or dyspnea	-4.6	-8.5, -0.6	-4.4	-8.8, -0.03	-2.2	-3.8, -0.6	-1.4	-3.0, 0.1
Asset ownership index								
Poorest	-3.3	-9.2, 2.6	-3.4	-9.9, 3.0	-4.2	-6.5, -1.9	-4.7	-7.1, -2.4
Poorer	0.1	-5.5, 5.8	-1.3	-7.4, 4.7	-0.8	-3.0, 1.4	-2.0	-4.2, 0.2
Middle	4.5	-0.3, 9.3	3.8	-1.4, 9.0	1.5	-0.3, 3.4	-0.3	-2.2, 1.6
Richer	0.3	-4.9, 5.5	-1.4	-7.1, 4.3	1.0	-1.0, 3.0	-1.1	-3.2, 1.0
HIV	-6.4	-15.0, 2.2	-8.0	-16.8, 0.8	-1.6	-5.0, 1.7	-1.6	-4.8, 1.6

Table S8 Legend

FEV_1: Forced expiratory volume in one second; FVC: Forced vital capacity; CI: Confidence interval; % predicted: percent predicted; % Δ: percent change

Reference categories for comparison: Smoking - lifelong non-smoker; Asset Index - Richest quintile

Table S9. Population prevalence of COPD among selected sub-groups; sensitivity analysis restricted to participants whose best spirometry trials were within 150mL

**Characteristic**	**Unweighted Study Sample (n = 397)**	***p* value**	**Population Weighted Estimates**	***P* value**
Prevalence	3% (n=10)		2%	
Age		0.81		0.31
18-29	1% (n=1)		0.2%	
30-39	3% (n=3)		0.9%	
40–53	3% (n=3)		0.6%	
54–93	3% (n=3)		0.4%	
Gender		1.0		0.34
Male	2% (n=4)		0.7%	
Female	3% (n=6)		1.3%	
Smoking history		0.49		0.76
Ever smoker	3% (n=4)		0.6%	
Never smoker	2% (n=6)		1.4%	
Asset index		0.32		0.67
Poorest	6% (n=4)		0.6%	
Poorer	3% (n=2)		0.3%	
Middle	2% (n=2)		0.3%	
Richer	1% (n=1)		0.3%	
Richest	1% (n=1)		0.6%	
Education		0.07		0.12
None	6% (n=4)		0.6%	
At least some 1˚ school	1% (n=3)		0.4%	
At least some 2˚ school	3% (n=3)		2.0%	
Occupation		0.47		0.69

Farmer	2% (n=6)		1.1%	
Other professions	4% (n=4)		1.1%	
HIV serostatus		1.0		0.60
HIV positive	0% (n=0)		0%	
HIV negative	3% (n=9)		2.1%	
Respiratory symptoms		0.10		0.01
Cough or dyspnea	5% (n=6)		1.4%	
None	2% (n=4)		0.7%	

Table S9 Legend

COPD: Chronic obstructive pulmonary disease; HIV: Human immunodeficiency virus; 1˚: Primary; 2˚: Secondary

Table S10. Unadjusted and adjusted correlates of lung function, not weighted for study participation

	FEV_1 (% predicted)				FEV_1:FVC (% predicted)			
	**Unadjusted**		**Adjusted**		**Unadjusted**		**Adjusted**	
**Characteristic**	***% Δ***	***95% CI***	***% Δ***	***95% CI***	***% Δ***	***95% CI***	***% Δ***	***95% CI***
Age, per year	0.24	0.15, 0.33	0.24	0.13, 0.36	0.03	-0.01, 0.06	0.05	0.00, 0.09
Female sex	-4.37	-7.65, -1.09	-5.00	-8.60, -1.39	-2.87	-4.09, -1.65	-3.18	-4.53, -1.84
Smoking	3.00	-0.54, 6.54	-3.95	-8.47, 0.57	-0.83	-2.17, 0.50	-2.40	-4.09, -0.71
Cough or dyspnea	-4.21	-7.58, -0.83	-2.80	-6.38, 0.78	-1.42	-2.69, -0.14	-0.45	-1.79, 0.89
Asset ownership index								
Poorest	-3.66	-8.85, 1.54	-0.75	-6.24, 4.74	-3.69	-5.62, -1.77	-2.65	-4.70, -0.60
Poorer	1.81	-3.37, 6.98	3.17	-2.31, 8.64	-2.05	-3.97, -0.13	-1.24	-3.28, 0.81
Middle	-0.37	-5.37, 4.64	1.49	-3.73, 6.70	-1.87	-3.73, -0.02	-1.48	-3.42, 0.47
Richer	1.53	-3.46, 6.51	2.08	-3.09, 7.26	0.56	-1.29, 2.40	0.11	-1.82, 2.04
HIV	-1.07	-8.81, 6.66	-1.78	-9.58, 6.02	0.17	-2.76, 3.09	0.14	-2.78, 3.05

Table S10 Legend

FEV_1: Forced expiratory volume in one second; FVC: Forced vital capacity; % predicted: percent predicted using National Health and Nutrition Examination Survey III prediction equations; % Δ: percent change; CI: Confidence interval; HIV: human immunodeficiency virus. Reference categories for comparisons: Smoking - lifelong non-smoker; Asset ownership index - Richest quintile
